# Immunological Aspects of Skin Aging in Atopic Dermatitis

**DOI:** 10.3390/ijms22115729

**Published:** 2021-05-27

**Authors:** Georgeta St. Bocheva, Radomir M. Slominski, Andrzej T. Slominski

**Affiliations:** 1 Department of Pharmacology and Toxicology, Medical University of Sofia, 1431 Sofia, Bulgaria; 2 Division of Rheumatology, Department of Medicine, University of Alabama at Birmingham, Birmingham, AL 35294, USA; radomir.slominski@gmail.com; 3 Department of Dermatology, Comprehensive Cancer Center, Cancer Chemoprevention Program, University of Alabama at Birmingham, Birmingham, AL 35294, USA; 4 Veteran Administration Medical Center, Birmingham, AL 35294, USA

**Keywords:** skin aging, atopic dermatitis (AD), skin immune responses

## Abstract

The cutaneous immune response is important for the regulation of skin aging well as for the development of immune-mediated skin diseases. Aging of the human skin undergoes immunosenescence with immunological alterations and can be affected by environmental stressors and internal factors, thus leading to various epidermal barrier abnormalities. The dysfunctional epidermal barrier, immune dysregulation, and skin dysbiosis in the advanced age, together with the genetic factors, facilitate the late onset of atopic dermatitis (AD) in the elderly, whose cases have recently been on the rise. Controversial to the healthy aged skin, where overproduction of many cytokines is found, the levels of Th2/Th22 related cytokines inversely correlated with age in the skin of older AD patients. As opposed to an endogenously aged skin, the expression of the terminal differentiation markers significantly increases with age in AD. Despite the atenuated barrier disturbances in older AD patients, the aged skin carries an impairment associated with the aging process, which reflects the persistence of AD. The chronicity of AD in older patients might not directly affect skin aging but does not allow spontaneous remission. Thus, adult- and elderly subtypes of AD are considered as a lifelong disease.

## 1. Introduction

Aging is a genetically determined natural phenomenon that leads to the progressive deterioration in physiological integrity, functional capacity, and morphological features of the organism [1]. While the skin has recognized stress response with its endocrine capabilities to respond to the environment [2,3], chronobiological aging is caused mainly by imbalances with endocrine functions, as well as hormonal decline with advancing age, leading to cumulative morphological and functional alterations of all organs and systems [3].

The aging of the human skin is also affected by the physiological maturing process that results in the phenotypic skin presentations [4,5]. The skin accumulates an excessive number of senescent cells, undergoes gradual loss of cellular functions, and could serve as a mirror of the first natural signs of aging. The main cellular perturbations in the skin inducing senescence are an inflammation and oxidative stress. It was found that the expression of Sirtuin (SIRT)-1 in human dermal fibroblasts is significantly reduced in advanced age [6]. Moreover, its downregulation results in accelerated fibroblast senescence [7]. Additionally, senescent skin cells are metabolically active and secrete pro-inflammatory cytokines, chemokines, proteases, and growth factors in a state called senescence-associated secretory phenotype (SASP) [8]. SASP contributes to functional decline of endogenously aged skin. The regenerative potential and homeostasis of the aging skin can be directly regulated by epigenetic mechanisms [9].

Skin exposome is described as the totality of exposures to both the environmental and internal factors over the human lifespan, thus influencing the skin aging [10,11]. The main environmental stressors are ultraviolet radiation (UVR) [5,12,13], tobacco smoking [14,15], and other pollutants and toxins [16,17], as well as microbial insults. They contribute to premature skin aging with wrinkling formation and pigmentation affecting predominantly the exposed areas of the body (face, neck, head, and hands). The most harmful factor from the environment that affects skin biology is UVR. Chronic sunlight exposure leads to superposition of the solar damage on the natural aging process resulting in chronic inflammation, impaired regenerative capacity, and carcinogenesis, associated with photoaging [18]. Some authors would consider smoking as a greater contributor to premature facial wrinkles than chronic sunlight exposure [19,20]. Additionally, smoking can decrease the serum levels of vitamin D_3_ [16], which is a well-known modulator of the immune system [21]. Air pollutants, persistent organic pollutants, and heavy metals can behave like endocrine-disrupting chemicals (EDCs) and indirectly may also cause vitamin D deficiency, whereas ozone and particulate matter (PM) can directly affect the cutaneous production of vitamin D [16]. Traffic-related air pollutants have an impact not only on skin aging but together with aeroallergens play a key role in the inflammatory response and clinical manifestation of atopic dermatitis (AD) [10].

The synergic effects of environmental and constitutive (internal) aging factors over the life course impair the epidermal barrier function with significant morbidity [22]. Skin in advanced age has insufficient perspiration and is susceptible to pervasive dryness and pruritus, infections, vascular complications (senile purpura, telangiectasia, etc.), and pigmentary changes (senile lentigines, etc.) [23].

## 2. Immunological Alterations in the Aging Skin

The process of aging is accompanied with immunological alterations, so that the immune system also undergoes senescence. The aged immune system is hyporesponsive to infection and vaccination [24] but exert an increased inflammatory response with a negative impact on healthy aging [25]. In advanced age, increased levels of inflammatory cytokines (IFN-γ, TNF-α, IL-1, IL-6) are commonly measured. The source of inflammatory mediators is the innate immune system. The imbalance between inflammatory and anti-inflammatory mechanisms in an aging phenotype causes a chronic low-grade inflammation, termed as “inflammaging” state [26,27]. Moreover, a reciprocal relationship between chronic inflammation and immunesenescence can be seen. The “inflammaging” is a result of both chronic antigen stimulation over the years and continued exposure to oxidative stress caused by free radicals and toxins production. These two factors contribute to the remodeling of the immune system and can modify the apoptotic potential of lymphocytes (Lym). Senescence of T-Lym is characterized by phenotypical and functional changes, including the loss of typical T-cell surface markers [28]. Because of both thymic involution (progressive decrease of new T-cell generation) in adults and chronic antigen stimulation, there is a reduction of the number and function of naïve Lym. The functional alterations of naïve T-Lym are mostly due to the shortening of the telomeres, reduced production of IL-2, and diminished ability for differentiation into effector-cells. During senescence, the number of memory T-Lym and CD8^+^ cells increase, and the CD4^+^/CD8^+^ T-cell ratio is inverted [29].

Humoral immunity also undergoes quantitative and qualitative (restricted antibody diversity) changes with advancing age [30]. Overproduction of certain cytokines (incl. IL-6—the cytokine of geriatricians) control B-cell differentiation and the immunoglobulin (Ig) production. During senescence, it occurs a reduction of IgM and an increase of IgG and IgA levels [31]. All of these changes in cellular and humural immunity led to a shift of the immune system towards an inflammatory and autoimmune mode, which facilitates the development of inflammatory reactions and allergies in advanced age. In fact, immunity appears to play a crucial role in the mechanisms of aging and the onset of age-related diseases [29].

With accelerating age, the profound remodeling of the immune system affects the skin, leading to a decline in its adaptive capability [32]. As a multifunctional organ in the human body, the skin plays an essential role in the local and systemic homeostasis [2,3]. Moreover, skin is considered as the largest immunological organ with its intricate network of resident immune cells (Lym, macrophages, mast, and dendritic cells) acting as defensive components in many processes, including prevention of infection [33,34].

Immunosenescence of the skin is accompanied by a deregulation of immune responses that could lead to impairment of the cutaneous immunological defense [35]. The resident immune cells in the epidermis that can initiate a local immune response are called Langerhans cells (LCs), but their number declines with age, and they show a reduced capacity to migrate to lymph nodes [36,37]. In addition, LCs in aging skin express less epidermal human β-defensin (HBD)-3, which is an important antimicrobial peptide (AMP) for a response to infections. The relative paucity of LCs results in impairment of cutaneous immunity of aged skin to microbial insults [38]. Providing evidence for this observation, a variety of bacterial infections is more common with accelerating age, and *Staphyloccocus aureus* (*S. aureus*) and β-haemolytic streptoccoci are most frequent skin pathogens in the elderly. Some fungal and viral infections also very commonly affect aged skin [39].

Dermal dendritic cells (DCs) are responsible for the antigen uptake and apoptotic function, but, in aged skin, they appear to be functionally impaired. The functional decline of aged DCs affects phagocytosis, migration, mitochondrial function, ability to stimulate T cells, and capacity to secrete interferon (IFN)-I and IFN-III [40,41]. Plasmocytoid DCs (pDCs) are a major source of IFN-I [42]. IFN-I plays an important role in DC differentiation and activation and promotes the differentiation of naïve CD4^+^ T-cells to polarized Th1 cells, thus increasing the cytotoxicity of both natural killer (NK) cells and CD8^+^ T-cells, and enhances antibody production [40,43]. Elderly pDCs exert a reduced ability to induce the secretion of IFN gamma (IFN-γ) in CD4^+^ and CD8^+^ T-cells [44]. IFN-III, secreted in a greater amount from monocyte-derived DCs (mDCs), plays a protective role in viral infections. Furthermore, it was shown that aged mDCs produced pro-inflammatory- (IFN-α, TNF-α, IL-6) instead of anti-inflammatory cytokines after the uptake of apoptotic cells [45]. The function of DCs is critical for the autoimmunity, initiation of inflammatory skin disorders, and clearing of infections in aging.

During aging, T-cells also undergo functional exhaustion, characterized by a decrease of functional activity and increase in inhibitory receptor expression, like the programmed cell death protein-1 (PD-1). The expression of PD-1 is found to be increased in both CD4^+^ and CD8^+^ T-cells in aged skin as compared to young skin, suggesting that older T-cells are more susceptible to inhibition via PD-1 signaling [46]. Most T-cells in the skin are resident memory T cells (Trm), generated as potent effector cells after exposure to antigen [47], and playing a vital role in control of skin infections [48]. An important regulatory cell type for skin immunity and homeostasis is CD4+ Foxp3 T-regulatory (Treg) cells [49]. The number of Foxp3^+^ Treg cells found is increased in older skin. Moreover, UV radiation can lead to induction of Foxp3^+^ Tregs that could suppress other immune cells via IL-10 production [50].

A recent research study established that the number of cutaneous mast cells (MCs), macrophages and CD8^+^ T cells was increased in chronobiologically aging skin by 40%, 44%, and 90%, respectively, whereas CD4^+^ T cells and neutrophils (Neu) were unchanged. MCs as innate immune cells contribute to altered skin homeostasis during aging with their impaired functionality and distribution. They accumulate in the papillary dermis of aged skin, where they are localized to macrophages and vasoactive intestinal peptide (VIP) positive nerve fibers [51].

Vitamin D_3_ is an essential component of a functioning immune system and is a protector of skin homeostasis [21,52,53,54,55,56,57,58]. Its production and metabolism at the skin contributes to barrier immunity and skin barrier function [21,53,54,59,60,61,62,63,64,65]. It can also be activated in immune cells to biologically active metabolites [66,67,68]. Vitamin D deficiency found in advanced age could decrease its immunosuppressive effects exhibited via modulation of epidermal LCs [69] and induction of Tregs [70,71,72].

## 3. AD in Adults and the Elderly

AD is a chronic inflammatory skin disorder affecting up to 20% of children and 10% of adults with higher prevalence in industrialized countries [73]. With the aging of the society, the incidence of AD among older adults is rising and represents 1–3% of elderly populations [74]. Severe AD can be seen in 4% [75] to 10–20% [76] of adult patients. While the prevalence of AD in adults (age < 60 years) is generally lower for males vs. females [76], male predominance is seen in elderly (age ≥ 60) with allergic AD [77].

AD is a heterogeneous disease with various phenotypes and endotypes [78], and with a predisposition to much allergic and nonallergic comorbidity [79,80,81]. Several phenotypes based on age of onset (pediatric vs. adult/elderly AD) [76,77], ethnic origin (European-American (EA) AD vs. African American, Asians, etc.) [82,83,84,85], clinical features, and therapeutic response, can be distinguished [78,86]. The endotype pattern of the disease includes acute and chronic [87], as well as extrinsic and intrinsic AD [88,89,90]. Extrinsic (allergic; atopy-related) AD represents about 80% of adult atopic patients and is associated with a high level of serum IgE. Elderly patients with this subtype show frequent allergic sensitization to airborne allergens (e.g., *Dermatophagoides farinae*, cedar and grass pollens, etc.) followed by food allergens [91]. The intrinsic (nonallergic) AD is a less common subtype (≈20%); however, it affects the elderly in an increased proportion [92]. Intrinsic AD has normal or low serum IgE levels, an absence of atopic background, and a lack of sensitization to environmental allergens but with the possibility to exhibit specific IgE against enterotoxins of *staphyloccus aureus* (*S. aureus*) and other microbial antigens [90,92,93]. An intermediate-allergic AD has also been described [77].

According to the most recent studies, AD cases in adults and elderly have been gradually increasing and were defined as adult- and elderly-AD subgroups, respectively [94]. The persistence of classic pediatric AD into the late adult phase is more often than previously estimated and accounts for 25% or more [95]. The more severe forms of IgE-allergic AD (incl. atopic march) initiated in early childhood exert more often persistent AD activity into adulthood [96,97,98]. The clinical course of the continuous type of early-onset AD varies from intermittent with periods of recurrence to chronic persistent AD. Adult- and senile onset of AD are also recognized. Extrinsic- and intrinsic AD in adults share similar clinical features. The main clinical manifestations include chronic or recurrent pruritic eczematous lesions and lichenification typically localized in the flexures, face (*atopic red face*), and neck (“*dirty neck*”) in adults, with the tendency toward the “reverse sign” (unaffected folds of the knees and elbows with lichenification around) in the elderly amid an overall dry skin. In about 20% of elderly patients with AD, eczematous erythroderma is seen [91].

**Figure 1:**
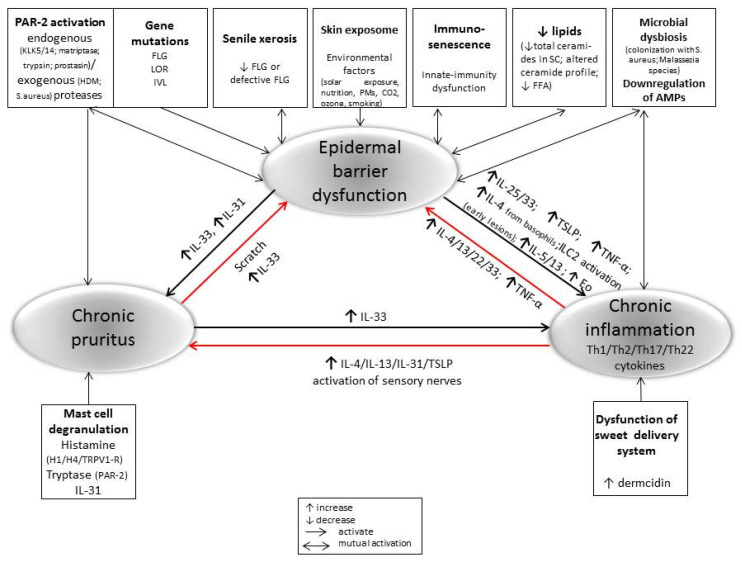
The crosstalk epidermal barrier dysfunction, inflammatory response, and chronic pruritus in the pathogenesis of perpetuate AD in older adults.

The pathogenesis of AD is complex and includes environmental and genetic factors, skin [99] and gut dysbiosis [100], dysfunctional epidermal barrier, and immune dysregulation [101]. All of these factors could serve as drivers that can promote and interact with the others, leading to chronic inflammation and a life-long illness in advanced age (Figure 1).

A skin barrier defect could be due to a dysregulation of corneal barrier proteins [102] and lipids in corneal layer [103,104], dysfunction of sweet delivery system [105], tight junction impairment [106], and overexpression of serine proteases (such as KLKs) [107].

It was found that the expression of terminal keratinocyte differentiation markers [filaggrin, loricrin] decreases with the age regardless of their mutation status [92,108]. Filaggrin (FLG), as an important structural protein for corneocytes in the outer epidermal barrier, influences cell differentiation and contributes to the natural hydration, antimicrobial protection, and cutaneous pH regulation. From the other hand, FLG can inhibit antigen formation by house dust mite (HDM)-derived phospholipase [109]. Additionally, up to 30% of AD patients show loss-of-function mutations in FLG, thus determining a more persistent course of the disease, but this is not crucial risk factors for the development of adult IgE-allergic AD [110]. Insufficiency of FLG causes skin dryness, elevated skin surface pH, colonization with *S. aureus*, and an allergen penetration in the skin [111].

Environmental and endogenous factors (e.g., alkalization of the skin) [112] can trigger a release of serine proteases that are capable of activating protease-activated receptors-2 (PAR-2) on keratinocytes and dermal nerves, thus leading to barrier disruption, cytokine release, intense itch, or NF-κB activation [113,114,115,116,117]. Additionally, mite cysteine proteases (HDM-derived antigens Der p1, p3, and p9) exert intrinsic protease activity and may accelerate the skin barrier impairment through PAR-2 activation, cytokine release, and Leu recruitment [118]. The scratching behavior due to intense pruritus can further lead to the physical damage of the epidermis and the beginning of the perpetual itch–scratch cycle.

The disrupted barrier function in aged skin allows penetration of pollutants, toxins, and allergens, resulting in both bacterial colonization and sensitization to allergens [119]. A reduction in microbial diversity through a domination of pathogenic *S. aureus* is a hallmark of skin microbiome in AD patients [39,120]. *S. aureus* with the ability to secrete proteases and to induce inflammation via super-antigens can also contribute to the AD pathogenesis, causing deepening of skin barrier defect and promoting a type 2 (Th2) immune activation [121]. Adult skin is also colonized with *Malassezia* fungal species which could trigger or exacerbate skin inflammation in AD [122].

## 4. Immunological Changes in AD Development

In AD, there exists a reciprocal relationship between the skin barrier dysfunction and the immune response, and both are necessary for the development of the disease. AD can be a primary immune-mediated disease with excessive T-cell activation and a reactive epidermal hyperplasia [123]. The late onset of AD (adult/elderly AD) might be linked to the underlying levels of inflammatory cytokines in the aged skin [26]. Sometimes, barrier defects can precede the immune activation that causes an inflammation and pruritus in AD. In the acute phase of the disease, disrupted epidermal barrier keratinocytes are triggered to produce and secrete alarmins IL-33, IL-25, and thymic stromal lymphopoetin (TSLP) to activate the type 2 innate lymphoid cells (ILC2s) and to promote a rapid recruitment of Th2 inflammatory cells and DCs in the early lesions. Activated ILC2s, both the natural (nILC2s) and inflammatory (iILC2s) cell types, produce a big amount of Th2 cytokines, such as IL-4 and IL-13 that results in eosinophil accumulation [79,124,125,126,127]. nILC2s are responsive to IL-33 [126], whereas iILC2s are responsive to IL25/TSLP [125]. IL-33 also stimulates basophils to produce IL-4, which drives a secretion of IL-5 and IL-13 from nILC2s. Additionally, IL-33 induces IL-31 and stimulates histamine release from MCs without an antigen, and both immune responses are causing pruritus [126]. In fact, Th2-Lym is responsible for the production of the potent pruritogenic cytokine IL-31, also called an “itchy cytokine”, whose level often correlates with the disease severity [128,129,130]. ILC2 activation triggered by IL-25 is essential for the expression of IL-13 in allergic cutaneous inflammation [125]. It was recently found that mite extracts, independent of sensitization, can induce IL-25 and IL-33 via the activation of Toll-like receptor (TLR)-1 and TLR-6 signaling [131].

In addition to the strong Th2 immune response driving an increased level of Th2 cytokines and chemokines (IL-4, IL-5, IL-13, IL-31, and CCL18) [132], the Th22 response (IL-22 and S100A proteins) is also characteristic for the acute AD lesions in adults [133,134]. As a result of scratching due to itching, the endogeneous TLR-4 ligands stimulate the production of IL-23 from keratinocytes [135]. IL-23 further activates IL-23R expressing DCs, which trigger an aryl-hydrocarbon receptor (AHR)-dependent Th22 immune response [136]. The activation of AHR stimulates a robust IL-22 expression on the affected keratinocytes in AD, implying a potential role of IL-22 as a promoter of epidermal hyperplasia and a contributor to the barrier defects [135,137]. The adult skin discloses an increased Th22 polarization, which presumably correlates with chronic immune stimulation over time. The main cell source, potentially producing IL-22 in AD, is circulating Th22- and CD4^+^/CD8^+^ T-cells, which often co-express IL-13 [138]. In in vitro studies, it is found that IL-22 directly upregulates IL-33 and TSLP [139], which could further amplificate the atopic skin inflammation. Moreover, Th2- (IL-4, IL-13) and Th22- (IL-22) derived cytokines downregulate the stratum corneum (FLG, LOR, IVL) [140,141,142,143] and tight junction protein expression (claudin) [144], thus inhibiting the expression of defensive AMPs in adult skin [145]. The lack of AMPs (catelicidin-LL37 and β-defensins) favors the contamination of virulent strains *S. aureus* that can interfere with the epidermal barrier [146]. Additionally, defects in TLR-2 also contribute to infection with *S. aureus* [147]. These findings in adults suggest that lesional atopic skin is always presented with a disrupted epidermal barrier.

In the Asian AD, multiple drivers of epidermal hyperplasia are found. This subtype of the disorder combines features of AD and psoriasis based on the association with an increased Th17 polarization and increased levels of IL-17 in lesional skin. Nonlesional skin of Asian AD patients is characterized by a high level of IL-22. Upregulated activation of Th2, Th17, and Th22 pathways in the skin of Asian adult patients exhibits similarity with pediatric EA AD [82,148,149]. IL-17 is the chemokine for Neu and T-Lym, which are significantly linked to the AD inflammatory response. Moreover, Neu infiltration is more consistently found in Asian versus EA AD [150].

Additionally, the Th17 axis shows an increasing involvement in intrinsic AD cases, which have similar Th2 activation to extrinsic endotype of EA AD in adults [88,92]. Th17-related products (IL-17A, MMP-12, PI3/elafin, and CCL20) are consistently upregulated in both acute and chronic intrinsic AD, but they are at lower levels than in psoriasis. IL-17A potentially decreases the expression of important genes involved in cellular adhesion and could downregulate FLG production/degradation, contributing to a barrier dysfunction [151]. IL-17A together with IL-22 regulates the transcription of S100A7/8/9 genes and may cause their upregulation in vitro [152,153]. Moreover, mRNA expression of the S100A is markedly increased in lesional skin of both endotypes (intrinsic and extrinsic AD) in comparison with nonlesional skin [88]. These S100A proteins can act as both inflammatory molecules and antimicrobial agents [137,154].

**Figure 2:**
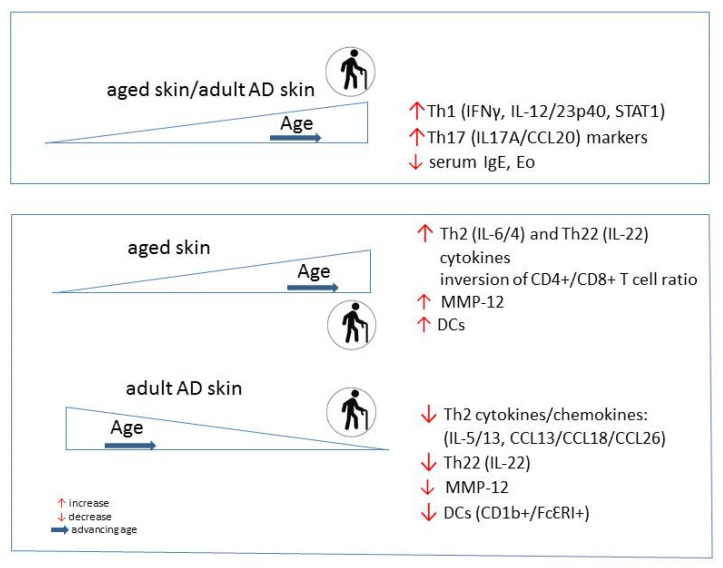
Skin-specific immunological properties of the healthy vs. AD population change during aging.

Chronic lesions of adult and elderly AD show a conversion of the dominated Th2 polarization to an involvement of multiple cytokine pathways with Th1 activation as an immune hallmark of these AD subtypes [133,155,156]. Th1 associated cytokines IFN-γ and IL-12 probably play a role for the chronicity of inflammation and for the apoptosis of keratinocytes [157]. An increased Th1 signaling together with Th17 activation is also a possible immunological state for the intrinsic endotype of AD, which becomes more frequent among older patients and is characterized by less FLG mutations [158]. The skin immunity skewing with a decrease of Th2/Th22 and an increase of Th1/Th17 activation is a unique immune feature in older atopic patients (Figure 2) [88,89,92]. The Th1 activation and higher production of IFN-γ negatively correlate with the disease severity possible due to suppression of Th2 proliferation and differentiation, and IgE production [158]. Moreover, IL-4 and its receptor (IL-4R) appear to decrease with chronicity of the disease. Because Th2 related cytokines can inhibit IL-17A production [159], the lowering of Th2 response could allow polarization toward Th17 cell cytokine activity. African American patients have a distinct attenuation of Th17/Th1 axes’ activation and low rates of FLG mutations [149].

The inflammatory infiltrates among lesional adult skin include a mixture of polarized CD3^+^/CD4^+^/CD8^+^ T-cells [156,160,161], DCs (CD111b+, FcɛRI^+^, CD206+) [162], MCs [163], and eosinophils. The number of MCs in atopic skin is increased compared to healthy ones, contributing to AD pathogenesis by production of inflammatory cytokines, like IL-17, IL-22, and IL-31 [163,164]. Skin infiltration with eosinophils is often associated with their increase in the blood and correlates with the disease severity in the acute phase of allergic AD [165]. Moreover, epidermal eosinophils in AD could express many Th2 products (IL-5, IL-13, IL-25, and CCL26) and thus may also contribute to skin inflammation [166].

Nonlesional skin of adult patients with chronic AD also shows immune abnormalities with an increased expression of Th2 (CCL22, CCL18, and IL-13), Th22 (IL-22), and Th1 (MX-1) immune cytokines and chronic T-cell expansion [167]. In addition, visibly unaffected skin shares similarities in terminal differentiation defects with atopic skin lesions, suggesting a need of systemic treatment of the severe chronic cases.

## 5. Skin Aging in AD

As age advances, skin develops functional impairments due to structural and morphological changes (such as 10–50% epidermal thinning, dermal remodeling, loss of elasticity, reduction in the number of sebaceous glands, etc.). Normal aged skin shows a marked decrease in corneal lipid content [168] and an impaired tight junction structure [169], synergically resulting in disrupted barrier function. The elevation of pH on the skin surface with age leads to a decrease of T-cell response to antigens, increasing its susceptibility to infections [170]. Furthermore, alkalization of the adult skin causes an impaired epidermal barrier as well, linked to activation of serine proteases and reduced activities of ceramide-generating enzymes [171,172]. In addition, senile xerosis [95], immunosenescence and neural degeneration can lead to chronic itch [173], and enhanced predisposition of late AD development that are significantly recognized in older adults [77,91].

Some characteristic immunological findings in AD in advanced age can be viewed in the normal skin aging process. These are the Th1 and Th17-related markers, which markedly increased with age, in both affected and unaffected skin in AD, similarly to healthy adult skin (Figure 2). However, in contrast with the skin of healthy adults, where Th2/Th22 related cytokines increase within normal aging, the levels of these cytokine inversely correlated with age in the skin of older AD patients. As a part of inflammaging of older skin, the increased cytokine milieu (Th2, Th22, Th1 and Th17) is associated with an increased number of epidermal DCs [137,174]. Conversely to normal skin, the number of inflammatory DCs (CD1b+, FcɛRI) is reducing in lesional and nonlesional atopic skin with age. Besides these findings, the expression of the marker of general inflammation, matrix metalloproteinase 12 (MMP-12) increases in normal skin as a function of age, contrary to its cutaneous expression in AD. Adult-age specific differences are also found in serum IgE level and number of eosinophils, which negatively correlated with age, causing a decreasing proportion of extrinsic AD among older adults [92].

In addition to the abnormalities in skin immunity, specific AD-associated changes in epidermal barrier can be seen in advanced age. As opposed to an endogenously aged skin, the terminal differentiation markers (e.g., FLG, LOR) expression significantly increases with age in AD. Other age-specific findings in AD are found in epidermal hyperplasia markers (Ki16, Ki67) [92]. The increased hyperplasia, which is characteristic for the atopic skin, diminishes in AD with age.

The chronological aging, presented by thinner, dry, pale skin with fine wrinkles, affects all skin areas and could be influenced from chronic inflammatory skin diseases like AD. Despite the ameliorated epidermal barrier disturbances through decreased hyperplasia and S100A expression, and increased terminal differentiation markers in older AD patients, the aged skin carries an intrinsic barrier compromise associated with the aging process. The intrinsic skin changes associated with normal aging (such as increased transepidermal water loss (TEWL), decrease physiological lipids, etc.) reflect the persistence of AD and skin lichenification in advanced age. With the multicytokine activated pathogenic circuits, the perpetuate AD in older patients might not directly affect skin aging but does not allow spontaneous remission. Indeed, AD in older adults can be considered as a lifelong disease, and there is a need in the future for a more personalized as well as a more targeted therapy.

## 6. Conclusions

The increasing incidences of AD in advanced age might be explained by the accumulation of the environmental stressors and their cumulative impact on the epidermal barrier functions. The impairment of the skin barrier functions exerts a reciprocal relationship with the skin immune system responses. These complex and context dependent interactions can induce and progress further immunological changes in both lesional and nonlesional skin in older patients with AD.

## Data Availability

Not applicable.

## Abbreviations

PAR-2	Protease-Activated Receptors-2
KLK	Kalikrein
HDM	House Dust Mite
FLG	Filaggrin
LOR	Loricrin
IVL	Involucrin
PMs	Particulate matters
AMPs	Antimicrobial peptides
FFA	Free Fatty Acids
IL	Interleukin
TSLP	Thymic stromal lymphopoetin
TRPV-1R	Transient receptor potential vanilloid-1 receptor
IFN	Interferon
MMP	Matrix metalloproteinase
STAT	Signal transducer and activator of transcription
CCL	Chemokine (C-C motif) ligand
